# Intelligent Thermoelectric Sensing with Sustainable Strain‐Hardening Geopolymeric Composites

**DOI:** 10.1002/smsc.202400520

**Published:** 2025-01-22

**Authors:** Jingming Cai, Yujin Yuan, Lin Pan, Zhiyang Pei, Yu Zhang, Xiang Xi, Neven Ukrainczyk, Eduardus A. B. Koenders, Linfeng Zhang, Y. X. Zhang, Jinlong Pan, Yifeng Wang, Wenjie Xie

**Affiliations:** ^1^ Key Laboratory of Concrete and Prestressed Concrete Structures of Ministry of Education Southeast University Nanjing 211102 China; ^2^ College of Materials Science and Engineering Nanjing Tech University Nanjing 210009 China; ^3^ College of Civil and Transportation Engineering Hohai University Nanjing 210098 China; ^4^ Institute of Construction and Building Materials Technical University of Darmstadt Franziska‐Braun‐Str 7 64287 Darmstadt Germany; ^5^ School of Transportation Southeast University Nanjing 211102 China; ^6^ School of Engineering, Design and Built Environment Western Sydney University Kingswood NSW 2751 Australia; ^7^ Materials and Resources Department of Materials and Earth Sciences Technical University of Darmstadt 64287 Darmstadt Germany

**Keywords:** pore structures, strain‐hardening geopolymeric composites, TE figures of merit (*ZT*), thermoelectric energy collections

## Abstract

Traditional thermoelectric (TE) building materials are limited in both performance and durability, requiring enhancements for effective energy solutions. This research investigates strain‐hardening geopolymeric composites (SHGC) for TE sensing applications. The influence of metal oxides on mechanical strength and TE characteristics is evaluated using isothermal calorimetry, computed tomography scanning, and focused ion beam (FIB)–transmission electron microscopy analysis. At ambient temperature, SHGC samples with MnO_2_ exhibit the highest Seebeck coefficient of 5470 μV K^−1^ with a measured power density of 29 μW m^−2^. Despite the presence of small strain cracks, the SHGC maintains about 69% of its original *ZT* value even after long‐term use. This discovery underlines the durability and efficiency of SHGC, demonstrating their potential for future infrastructure applications. The cost‐effectiveness, temperature‐sensing abilities, and environmental advantages of SHGC make them well suited for large‐scale smart applications.

## Graphical abstract

Strain‐hardening geopolymeric composites (SHGCs) have been developed to combine thermoelectric sensing with improved structural resilience, crack resistance, and durability. By incorporating polyvinyl alcohol fibers and metal oxides, SHGC achieves high Seebeck coefficients and superior strain‐hardening behavior, ensuring effective crack control. These combined properties make SHGCs well suited for applications in temperature sensing, energy harvesting, and sustainable smart building systems.

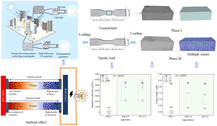

## 1 Introduction

**Figure 1:**
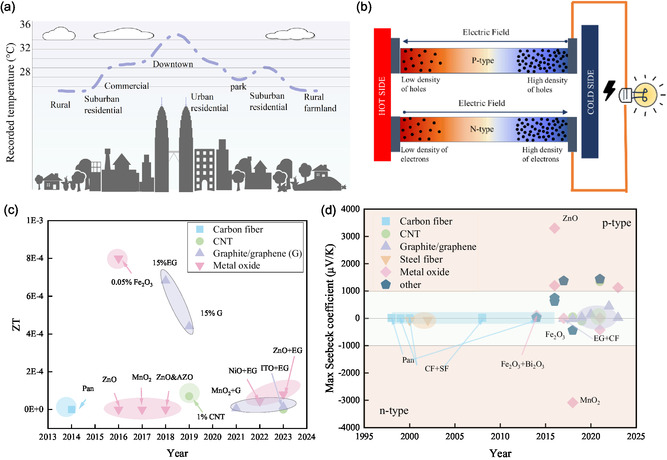
TE sensing property. a) Urban heat island effect. b) Power generation mode of TE materials based on the Seebeck effect. c) The timeline for *ZT* of the cement‐based TE materials with different additives. d) The timeline for the Seebeck coefficient of the composites.^[^ ^13^, ^48^ ^]^

The world is facing a severe energy crisis marked by a heavy reliance on nonrenewable fossil fuels. Currently, fossil fuels such as oil, coal, and natural gas account for ≈80% of the world's energy.^[^ ^1^ ^]^ This is because proven oil reserves will run out in less than 50 years if production holds up at the current rate.^[^ ^2^ ^]^ Over the past century, global temperatures have risen by nearly 1.5 °C; metropolitan areas, in particular, suffer from the urban heat island effect (**Figure** **1**a), in which buildings, roads, and other infrastructure absorb and re‐emit more heat than in natural environments.^[^ ^3^ ^]^ Rising energy demand for cooling results from higher temperatures which in turn amplifies carbon emissions from fossil fuel consumption, thus contributing to global warming.^[^ ^4^ ^]^ Moreover, buildings account for about 30% of the world's total energy consumption and 60% of its electricity usage, with heating, air conditioning, and other appliances making up the majority of this demand.^[^ ^5^ ^]^ In this sense, effectively managing smart buildings becomes important to optimizing indoor comfort while reducing energy consumption.

Thermoelectric (TE) technology is under investigation for infrastructure, particularly in buildings and roads, amid current energy issues and the urban heat island effect.^[^ ^6^ ^]^ TE technology can generate electricity from the temperature gradient (Figure 1b).^[^ ^7^ ^]^ TE generators harness waste heat, converting temperature gradients into electricity to power sensors autonomously and reducing battery dependence and energy demand.^[^ ^8^ ^]^ TE fabrics have been studied to address challenges such as durability and environmental stability, and developments have enabled the design of self‐powered wearable systems for applications like health monitoring and motion detection.^[^ ^9^ ^]^ This technology is particularly appealing for buildings, as it can detect temperature differences between indoor and outdoor environments to intelligently control conventional heating, ventilation, and air conditioning (HVAC) systems.^[^ ^10^ ^]^ The more effective operation of HVAC systems made possible by this real‐time detection improves indoor comfort, energy economy, and sustainability.^[^ ^11^ ^]^ However, using commercial TE modules in buildings presents difficulties because of low efficiency, uncertain durability, and expensive costs.^[^ ^12^ ^]^ To address these challenges, research has focused on the development of cement‐ and geopolymer‐based TE composites, which combine structural strength with temperature‐sensing capabilities.

According to Figure 1c,d, the timeline for TE materials in the cement mixture generally shows lower *ZT* and Seebeck values.^[^ ^13^ ^]^ Although TE cement composites provide a cost‐efficient solution for temperature‐sensing buildings, low TE performance, mechanical problems, and brittleness affecting sensing efficiency remain.[^13^, ^14^] Since silicon and aluminum possess a more excellent homogeneous molecular structure than conventional cement‐based materials, geopolymers provide superior TE performance and facilitate sensing temperature differences.^[^ ^15^ ^]^ With their ultimate tensile strength only about 10% of their compressive strength, geopolymers exhibit brittle fracture behavior similar to cement under tensile conditions. If not sufficiently optimized, the intrinsic brittleness of geopolymers could compromise the long‐term durability of buildings. In this case, strain‐hardening geopolymeric composites (SHGC) have shown notable promise as both temperature‐sensing and crack‐resistant materials.[^15^, ^16^] Although several kinds of fibers have been tested to improve the ductility of geopolymeric composites, ordinary fiber‐reinforced geopolymers still exhibit traits of primary crack propagation and strain softening.^[^ ^17^ ^]^ PVA fibers have been selected for use in SHGC materials, considering ultra‐high molecular weight polyethylene fibers’ relatively high cost and lower long‐term durability.^[^ ^18^ ^]^

Smart buildings represent one of the most exciting uses for SHGCs as they help architects and engineers develop more efficient insulation and energy‐saving techniques, thus improving the sustainability of buildings.^[^ ^19^ ^]^ The self‐sensing features of smart buildings are also well‐suited for environmental monitoring in remote regions, such as weather stations and ocean monitoring systems.^[^ ^20^ ^]^ The device uses the thermal gradient to automatically generate electricity, allowing continuous sensing without external power sources.

In this work, polyvinyl alcohol (PVA) fibers were introduced to lower the intrinsic brittleness of geopolymer composites (GCs). Considering the natural TE characteristics of metal oxides and their great compatibility with calcium–aluminosilicate–hydrate gel,[^14^, ^21^] additives including MnO_2_, Fe_2_O_3_, ZnO, and carbon black were incorporated to improve the sensing characteristics of SHGC. The Seebeck coefficient was calculated using a controlled temperature gradient, and the electrical and thermal conductivity were measured using AC impedance spectroscopy and a self‐designed apparatus, respectively. Furthermore, compressive strength tests were used to confirm the mechanical integrity of the composites. A comparative analysis of mechanical strength and fracture control capabilities under tensile stress demonstrated superior performance in smart structure applications.

## 2 Mechanical Properties

For the one‐part GC samples, the letter “G” represents the geopolymer samples. Various oxide additives are differentiated based on their chemical elements. The number following the dash represents the additive content in weight percent. The letter P indicates the presence of PVA fiber. For example, the GMn‐5‐P sample contains PVA fibers and 5 wt% MnO_2_. Compressive strength tests were conducted to assess the performance comprehensively to evaluate load‐bearing capacity, tensile tests were performed to observe cracking behavior, and fractal dimension analysis was used to quantify the complexity of crack distributions.

The average compressive strengths for all specimens are displayed in **Figure** **2**a. Compared to the pure solid precursor (G‐0), the compressive strength of the GMn‐5 increased by 4.1%. Similar observations were noted for SHGCs. Therefore, it can be inferred that the inclusion of MnO_2_ positively influences the compressive strength of geopolymer when cured at ambient temperature. The GCs exhibit a maximum compressive strength of 65.6 MPa after 14 days of curing, which is considered suitable for engineering purposes.^[^ ^22^ ^]^ According to Figure 1a, the greater number of microfractures of SHGC samples suggests that the inclusion of fibers effectively prevents spalling and crushing. Although adding fibers reduces compressive strength, specimen GMn‐5‐P shows equivalent strength to specimen GMn‐5. Likewise, the addition of Fe_2_O_3_ was seen to strengthen the matrix by acting as a nanofiller similar to MnO_2_.^[^ ^23^ ^]^ This boost can be attributed to the capacity to fill vacancies, improving its density and promoting strong interfacial bonding. A rise in MnO_2_ content corresponds, within a threshold, with a rise in compressive strength.

Figure 2Mechanical properties and fractal dimension. a) Compressive strength and typical failure modes. Data are expressed as means *s* ± standard deviation (SD) (*n* = 3) b) Tensile stress–strain curves of SHGC. Data are expressed as means s ± SD (*n* = 3). c) Micromorphology of the specimen. d) Macrofailure fractal dimension of SHGC by box‐counting method. e) Porous 3D structure based on computed tomography (CT) scan.

**Figure:**
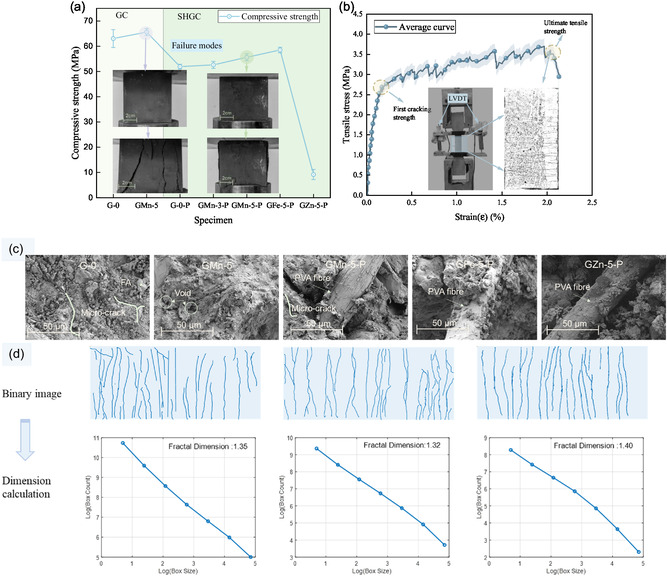

**Figure:**
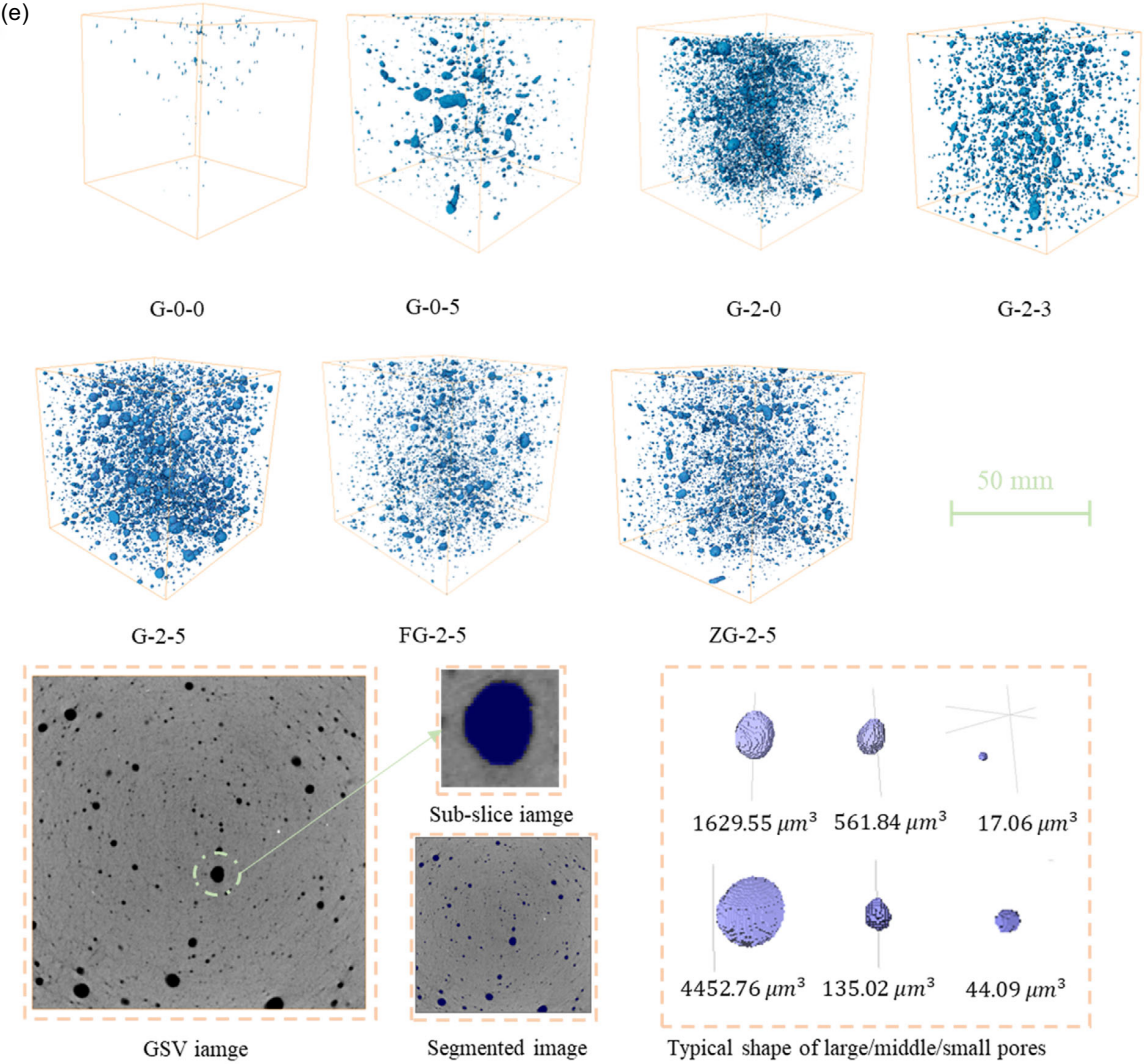

However, the GZn‐5‐P samples exhibited compressive strength of less than 10 MPa. This considerable reduction can be due to the amphoteric nature of ZnO, which reacts with the alkaline geopolymer environment, altering the matrix structure and lowering strength (Figure S2, Supporting Information). Regarding the long‐term service characteristics of a structure, the SHGC sample displayed strain‐hardening properties and a densely distributed pattern of multiple cracks after the uniaxial tensile test (Figure 2b). The initial fracture strength is 2.5 MPa, the tensile strength reaches 3.5 MPa, and the maximum tensile strain is as high as 3%. The microscopic morphology of the samples is shown in Figure 2c. The accumulation of low‐crystalline calcite in certain areas naturally results in uneven expansion, potentially causing the formation of significant microcracks within the geopolymer.^[^ ^24^ ^]^ Moreover, the presence of PVA fibers within the geopolymer matrix demonstrated a strong ability to bridge gaps.^[^ ^25^ ^]^

SHGC, a novel composite material featuring 3D, randomly distributed reinforcing fibers and numerous interfaces, typically undergoes a complex multicracking process as part of its deterioration. The FracLab toolbox calculates the fractal dimension (Section S3, Supporting Information). The fractal dimension parameters typically observed on the surface of ordinary concrete specimens tend to range between 1.1 and 1.3.^[^ ^26^ ^]^ Conversely, SHGCs generally exhibit values within a slightly higher range of 1.3–1.4 (Figure 2d). This suggests that the fracture distributions of SHGCs are more intricate than previously thought. Compared to GCs, SHGCs exhibit a higher porosity, which may lead to more pronounced multiple cracking behavior (Figure 2e).^[^ ^27^ ^]^ The microstructural data also revealed a distribution in which SHGC samples exhibited a greater porosity and a higher count of substantial pores (Section S4, Supporting Information).

## 3 Thermoelectric Properties

### 3.1 Seebeck Coefficient and Electrical Conductivity

**Figure 3:**
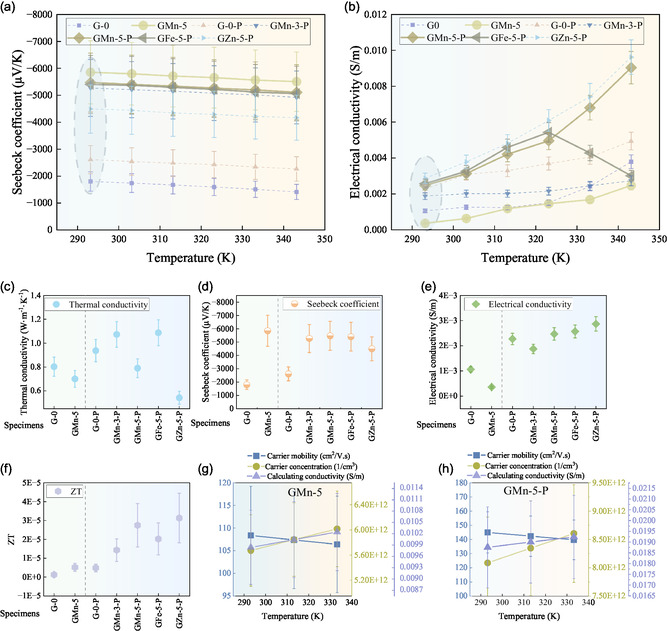
TE properties. a) Summary of the Seebeck coefficient, which has an uncertainty of about ±20%. b) Summary of electrical conductivity, which has an uncertainty of about ±10%. c–f) Thermal conductivity, electrical conductivity, Seebeck coefficient, and *ZT* at ambient temperature exist with uncertainties of about ±10, ±10, ±20, and ±42%, respectively. g, h) Carrier concentration and mobility in the Hall effect tester for the GMn‐5 and GMn‐5‐P samples, which has an uncertainty of about ±10%.

As presented in **Figure** **3**a, it is worth noting that geopolymers that added metal oxides exhibit negative potential, which is dependent on the energy differential.^[^ ^28^ ^]^ The highest values were observed for samples GMn‐5, which did not contain PVA fibers, as well as GMn‐5‐P and GFe‐5‐P, both of which were incorporated with PVA fibers (Figure 3a). Relative to sample G‐0, the Seebeck coefficient of sample GMn‐5, with added MnO_2_, was three times higher and reached a mean value of 5850 μV K^−1^ at 293 K. For the SHGCs, the Seebeck coefficient of sample GMn‐5‐P attained 5470 μV K^−1^. The Seebeck coefficient varied by roughly 10%, while electrical conductivity varied by ≈5%. Substituting MnO_2_ with Fe_2_O_3_ resulted in a slight decrease in the Seebeck coefficient, yet it still achieved a high value of about 5400 μV K^−1^ for GFe‐5‐P. A plausible explanation for this could be that the inclusion of the oxides results in a localized increase of *g(E)* within a relatively narrow energy range.[^21^, ^29^] Equation (1) and (2)^[^ ^30^ ^]^ indicate this phenomenon, which is presented below for further clarification.

(1) $$S=\frac{\pi^{2}}{3}\frac{k_{B}}{q}k_{B}T{\left\{\frac{1}{n}\frac{dn\left(E\right)}{dE}+\frac{1}{\mu }\frac{d\mu \left(E\right)}{dE}\right\}}_{E=E_{F}}$$

(2) n(E)=g(E) f(E)

where *k*_B_ is the Boltzmann constant, *n*(*E*) represents the carrier density at the energy level *E* under consideration, *q* is the charge of the carrier, *μ*(*E*) *is* the mobility, *g*(*E*) is the density of states, and *f*(*E*) is the Fermi function.

The SHGCs demonstrate superior electrical conductivity relative to the GCs, likely due to enhanced ionic conductivity caused by the porous structure^[^ ^31^ ^]^ (Figure 2e and Section S4, Supporting Information). Subsequently, the electrical conductivities of the oxide‐added samples abide by the following order: ZnO, Fe_2_O_3_, and MnO_2_, although the differences are not obvious. The relatively higher conductivity of GZn‐5‐P could potentially be attributed to the formation of a zincate ion (Figure S2, Supporting Information).^[^ ^32^ ^]^ In contrast, the electrical conductivity of GFe‐5‐P behaves differently, which might be due to its crystal structure. GFe‐5‐P exhibits a higher degree of crystallinity, as indicated by the absence of C=C double bond vibrations and the strong Fe—O bond signal in the Raman spectra, alongside sharp diffraction peaks in X‐ray analysis, which can reduce ionic mobility and lead to lower electrical conductivity compared to more amorphous structures (Figure S4, Supporting Information).

In the specimens GMn‐5 and GMn‐5‐P, as the temperature increased, the carrier concentration rose while the mobility decreased (Figure 3g,h). The calculated electrical conductivity showed an upward trend consistent with the increased thermal excitation of charge carriers, though discrepancies with the measured values were noted due to carrier scattering at phase interfaces and pore walls. These scattering effects, more prominent in SHGCs due to their higher porosity, may have contributed to the observed deviations.^[^ ^33^ ^]^ In the study, GMn‐5‐P exhibited a higher carrier concentration and mobility compared to GMn‐5. It is hypothesized that the incorporation of PVA fibers may have facilitated the adsorption of carbon black, thereby promoting percolation pathways for charge transport (Figure S1a, Supporting Information).^[^ ^34^ ^]^

**Figure 4:**
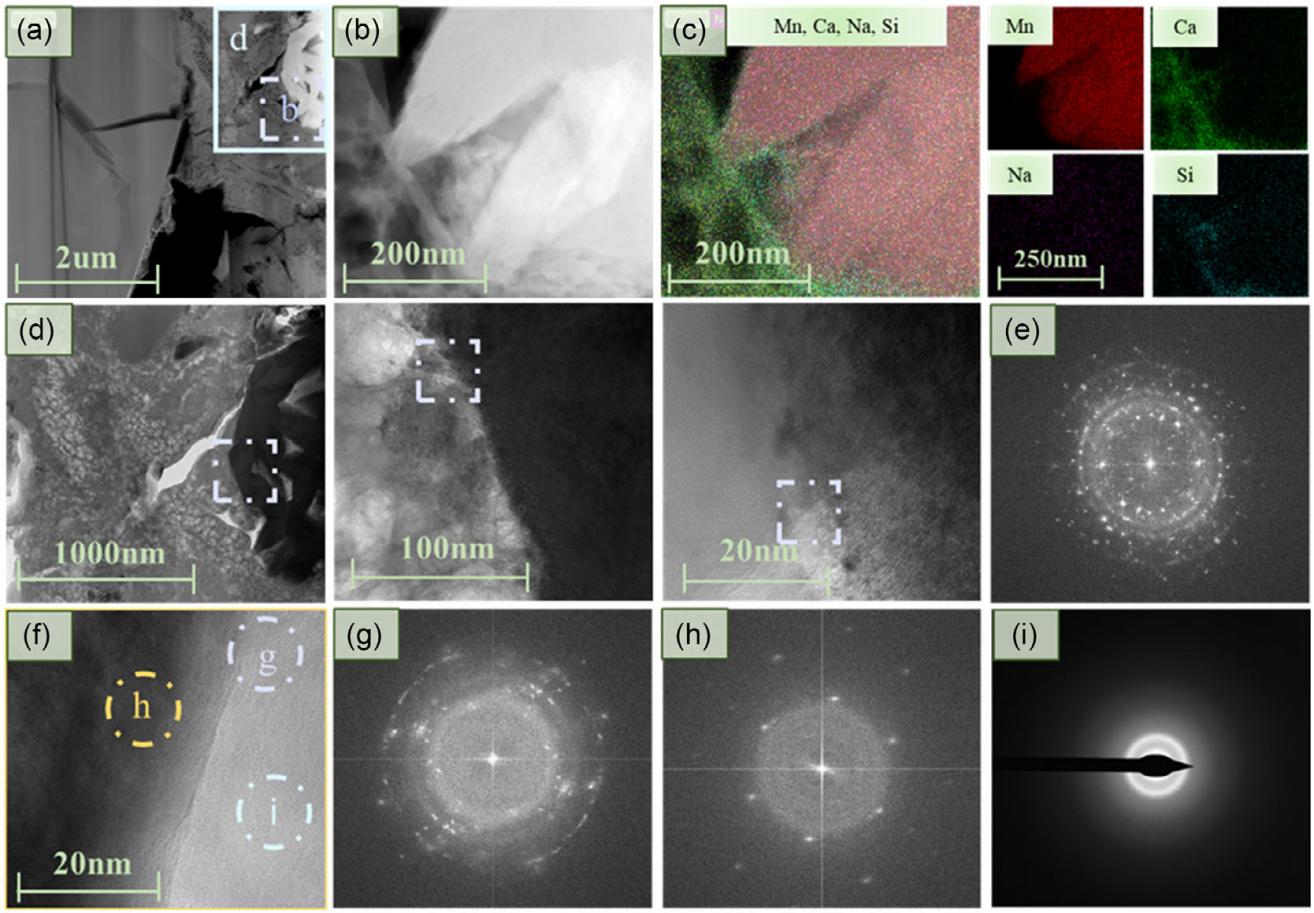
Micro/nanostructural characteristics of the phase boundaries of MnO_2_ and C–S–H. a) The morphology of the target area beneath the platinum cover. b) The HAADF magnified image from the square area in (a). c) EDS maps for overlap as well as Mn, Ca, Na, and Si. d) Different scales of TEM images magnified from the blue square area in (a). e) FFT pattern taken from the purple area in (d). f) Gel interface of the interested area. g) FFT pattern taken from the purple oval in (f), implying a typical polycrystalline state at the interface. h) FFT pattern taken from the orange area in (f), indicating ordered MnO_2_ lattice. i) The FTT pattern of C–(N–) A–S–H gel phases taken from the blue area in (f).

The nanostructural characteristics of the GMn‐5‐P sample were further investigated using comprehensive transmission electron microscopy (TEM) and energy‐dispersive spectroscopy (EDS) (**Figure** **4**). These subregions exhibit inherent morphological differences at the nanoscale, allowing for the clear distinction of the initial boundaries of MnO_2_ (Figure S5, Supporting Information). Figure 4a presents a high‐angle annular dark field scanning electron microscope (HAADF) micrograph, while Figure 4b,c provides an enlarged view of the purple area, displaying the major elemental mappings of Mn, Ca, Na, and Si across the three subregions. As depicted in Figure 4a, stress concentrations arise at the interfaces with temperature variations, leading to the formation of microcracks or weak zones.^[^ ^35^ ^]^ The region under HAADF mode exhibits a significant amount of dark areas, suggesting the presence of pores. The boundary areas reveal an interlayer rich in Ca and Si, the primary components of the highly disordered CSH gel phase. Additionally, sodium ions exhibit a relatively uniform distribution.

In this subregion, the boundary regions exhibit irregular fast fourier transform (FFT) diffraction spots, likely due to the dispersion of hydrotalcite crystals (Figure 4d,g).^[^ ^36^ ^]^ These irregularities often suggest that the material contains multiple crystalline domains with varying orientations. The interfacial barriers may act as energy filters, scattering low‐energy carriers, which significantly increase the Seebeck coefficient.^[^ ^37^ ^]^ However, they may also cause electron scattering, resulting in a reduction in electrical conductivity.^[^ ^7^ ^]^ A well‐ordered lattice structure is evident in MnO_2_ (Figure 4h), and the large Seebeck coefficient is likely closely associated with the surface density at the Fermi energy level.[^21^, ^29^] In contrast, the foil‐like morphology of the hydration products corresponds to the CSH gel phase reported in the literature.^[^ ^38^ ^]^ The absence of lattice fringes and distinct diffraction spots in the FFT pattern confirms that the C—(N—) ASH gel phase formed in this region is largely disordered (Figure 4i).

The migration and transport of ions are crucial factors influencing the electrical conductivity of geopolymer.^[^ ^39^ ^]^ For the SHGC‐MnO_2_, there was an initial decrease in electrical conductivity, followed by an increase as the MnO_2_ content increased. This phenomenon may be attributed to these reasons: high‐porosity samples possess a more complex and interconnected network of pore channels that facilitate ionic migration. Among these, the sample GMn‐5‐P exhibited the highest porosity, with G‐0‐P coming in second (Figure S3, Supporting Information). Since ionic migration within these channels is relatively facile, it promotes the enhancement of electrical conductivity (Section S7, Supporting Information).

The specific impedance reduces consistently as the dose of metal oxide increases (Figure S6, Supporting Information). However, the dosage and species of the additive influence the specific direction of this change. Despite a 7‐day curing period of the GCs and SHGCs, significant quantities of conductive ions, including K^+^, Na^+^, and OH^−^, remain present.^[^ ^40^ ^]^ After depolarization, the *ZT* values under AC impedance show a similar trend to those measured under DC conditions. However, the numerical values experience an order‐of‐magnitude increase. The capacitive characteristics of the porous structure within the geopolymer samples inextricably link to this phenomenon.

### 3.2 Thermal Conductivity

The thermal conductivities of the geopolymer were tested using a self‐designed device at ambient temperature (Figure 3c). The addition of admixture to the geopolymer has a noticeable influence on the thermal conductivity. The values for GMn‐5 and GMn‐5‐P were 0.70 and 0.79 W m^−1^ K^−1^, respectively. With an increasing MnO_2_ content, a decreasing trend in thermal conductivity was observed. The abrupt increase in thermal conductivity for GMn‐3‐P compared to G‐0‐P may be attributed to a relatively lower porosity structure, which enhanced thermal conductivity (Figure 3e). Unlike MnO_2_, the addition of Fe_2_O_3_ enhances the conductivity of the SHGCs. It was also found that the thermal conductivity of GMn‐5 is 12.8% less than the sample containing PVA fiber. Although the literature mentions that adding PVA fibers can lead to a reduction,^[^ ^41^ ^]^ the addition of carbon black, coupled with its adherence to PVA fibers, has resulted in an enhancement.^[^ ^42^ ^]^ The slightly low thermal conductivity of the geopolymer system can be related to the presence of various lattice structures associated with distinct stages (Figure 4d). Regional lattice distortions may influence the scattering of short‐wavelength phonons, and the presence of observable grain and phase boundaries contributes to the effect, resulting in a drop in thermal conductivity.^[^ ^43^ ^]^

### 3.3 ZT

The enhancement of *ZT* in geopolymer samples can be achieved by selecting appropriate additives and optimizing their concentrations.^[14a]^ Notably, adding substances like MnO_2_ and Fe_2_O_3_ to geopolymers leads to a significant increase in the Seebeck coefficient, and the amount of additive also affects this coefficient. Although an enhancement in the Seebeck coefficient might coincide with a reduction in electrical conductivity, optimal TE performance can be attained through strategically co‐adding with conductive materials, facilitating an equilibrium. Furthermore, introducing defects may alter the electronic environment at the Fermi level, influencing TE properties. The *ZT* values for SHGC‐Oxide all exceed 2 × 10^−5^, with GZn‐5‐P exhibiting a higher value, recognized as a potentially attractive n‐type TE material.^[^ ^44^ ^]^ However, it is unsuitable for structural applications due to its lower compressive strength and the incomplete geopolymerization reaction (Figure S2, Supporting Information). Compared to GFe‐5‐P, the value of GMn‐5‐P is 0.25 times greater, reaching 2.74 × 10^−5^. Two conductivity modes may exist in porous materials, namely electronic and electrolytic conduction. It is advisable to use alternating current to address the issue of electric polarization. The relationship extends to the *ZT* value in the AC state for SHGC, and the values are approximately tenfold those in the DC state (Figure S6, Supporting Information).

### 3.4 TE Properties of Cracking Work

**Figure 5:**
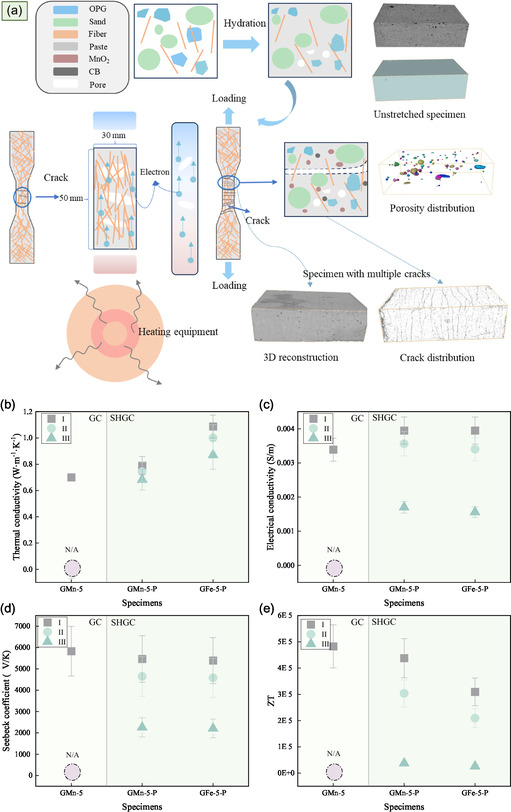
Correlation of *ZT* coefficients after SHGC cracks appear. a) Schematic diagram of SHGC hydration process and tensile test. b) Thermal conductivity with an uncertainty within ±10%. c) Electrical conductivity with an uncertainty within ±10%. d) Seebeck coefficient with an uncertainty within ±20%. e) *ZT* with an uncertainty within ±42%. Phase I is indicative of the specimen prior to the application of strain; Phase II marks the initial emergence of cracks; and Phase III reflects the condition at 80% of the ultimate tensile load. In the Phase II, a slight decreasing trend in electrical conductivity was observed, but the magnitude did not exceed 10%.

The method employed for tensile testing of SHGC posthydration is depicted in **Figure** **5**a, with thermal conductivity, Seebeck coefficient, electrical conductivity, and *ZT* shown in Figure 5b,e. Phase I represents an untensioned 30 × 50 mm prism from the dog bone specimen. Phase II marks the initial appearance of cracks. Phase III corresponds to the state at 80% of maximum tensile strength. In the absence of tensile strain (Phases I), electrical conductivity exhibits a relative increase in comparison to that of the abovementioned cylindrical specimens, which might be attributed to the reduction in defect density due to size effects.^[^ ^45^ ^]^ The thermal conductivity, measured using the plane heat board method, showed little variation, while the electrical conductivity exhibited a slight decrease during Phases II and III. The Seebeck coefficient remained at ≈85% in Phase II and nearly halved in Phase III. These cracks reduced the formation of thermal gradients and altered the migration paths of charge carriers. Nevertheless, even after the formation of cracks, the *ZT* could still maintain ≈69%, highlighting the remarkable perception ability of the smart structure under strained conditions.

The module GMn‐5‐P has an average Seebeck coefficient of 5465 μV K^−1^ and electrical conductivity of 0.004 S m^−1^. The mean power output can be determined using Equation (3).^[^ ^46^ ^]^

(3) $$P=\frac{S^{2}{\left(T_{h}-T_{c}\right)}^{2}}{4R}$$

Utilizing this equation, the output power of each cylinder is calculated to be ≈0.057 μW. When positioned adjacent to the cracks, the output power per square meter reaches 29 μW m^−2^, which is comparable to the power required by an intelligent sensing system.

## 4 Conclusion

In summary, the addition of MnO_2_ enhanced the compressive strength and strain‐hardening properties of the samples, with SHGCs exhibiting a high Seebeck coefficient and *ZT* values exceeding 2 × 10^−5^. The *ZT* values demonstrate the resilience of SHGCs, as they maintained 69% of their initial value even after long‐term usage. Although SHGCs exhibit less conversion efficiency than commercial TE modules, their environmental advantages and economy make them appropriate for large‐scale applications. Besides, the output power per square meter reached 29 μW m^−2^, indicating potential applications in temperature sensation. The self‐detection capability of SHGCs can support improved thermal management, energy efficiency, and sustainability in HVAC operations. Further optimization could enhance their role in advancing energy‐efficient technologies for the construction industry.

## 5 Experimental Section

5.1

5.1.1

##### Materials

The mixing and molding process is shown in Section S8, Supporting Information.

##### Isothermal Calorimetry Test

To examine the influence of additives (MnO_2_, Fe_2_O_3_, and ZnO) on the geopolymerization of SHGC, the calorimetry tests were conducted. These tests utilized an eight‐channel TAM Air isothermal calorimeter, which operated at 20 °C. Initially, preweighed one‐part geopolymer composite powder and water were stored separately in a glass ampoule and plastic injector. These containers were then positioned inside the calorimeter for 12 h to reach thermal equilibrium. Subsequently, water was introduced into the glass ampoule, followed by a 5‐minute mixing period using a mini stirrer. Throughout the process, heat flow was continuously monitored and recorded by data loggers.

##### Mechanical Test

The compressive and tensile strengths were evaluated using an electronic universal testing machine, following the standard JSCE‐2004.^[^ ^47^ ^]^

##### Thermal Conductivity Measurement

Details of the room temperature thermal conductivity test method are shown in Section S9, Supporting Information.

##### Seebeck Coefficient

Cylinders with dimensions of 50 mm in diameter and 100 mm in height were employed for the Seebeck coefficient test (Section S10, Supporting Information).

##### Electrical Conductivity Measurement

To analyze the electrical behavior of the samples, both DC conductivity and AC conductivity were analyzed, and Section S11, Supporting Information, provides further specifics.

## Conflict of Interest

The authors declare no conflict of interest.

## Author Contributions

**Jingming Cai**: data curation: (lead); formal analysis: (lead); investigation: (lead); methodology: (lead); validation: (lead); writing—original draft: (lead); writing—review & editing: (lead). **Yujin Yuan**: data curation: (lead); formal analysis: (equal); investigation: (equal); writing—original draft: (lead). **Lin Pan**: data curation: (supporting); formal analysis: (supporting); investigation: (supporting); methodology: (supporting); writing—review & editing: (supporting). **Zhiyang Pei**: data curation: (supporting); formal analysis: (supporting); investigation: (supporting); methodology: (supporting). **Yu Zhang**: data curation: (supporting); formal analysis: (supporting); investigation: (supporting); methodology: (supporting). **Xiang Xi**: data curation: (supporting); formal analysis: (supporting); investigation: (supporting); methodology: (supporting). **Neven Ukrainczyk**: formal analysis: (supporting); investigation: (supporting); methodology: (supporting); writing—review & editing: (supporting). **Eduardus A.B. Koenders**: resources: (supporting); supervision: (supporting); writing—review & editing: (supporting). **Linfeng Zhang**: formal analysis: (supporting); investigation: (supporting); methodology: (supporting); resources: (supporting). **Jinlong Pan**: conceptualization: (lead); funding acquisition: (lead); supervision: (lead); writing—review & editing: (supporting). **Y. X. Zhang**: investigation: (supporting); supervision: (supporting); writing—review & editing: (supporting). **Yifeng Wang**: conceptualization: (equal); resources: (supporting); supervision: (supporting); writing—review & editing: (equal). **Wenjie Xie**: conceptualization: (equal); formal analysis: (equal); supervision: (equal); writing—review & editing: (equal).

## Supporting information

Supplementary Material

## Data Availability

The data that support the findings of this study are available from the corresponding author upon reasonable request.

## References

[1] M. K. G. Deshmukh , M. Sameeroddin , D. Abdul , M. Abdul Sattar , Mater. Today: Proc. 2023, 80, 1756.

[2] E. V. London , WORLD ENERGY COUNCIL 2021.

[3] a) C. McGlade , P. Ekins , Nature 2015, 517, 187 25567285 10.1038/nature14016

[4] D. Welsby , J. Price , S. Pye , P. Ekins , Nature 2021, 597, 230. 34497394 10.1038/s41586-021-03821-8

[5] a) G. Tumminia , F. Guarino , S. Longo , D. Aloisio , S. Cellura , F. Sergi , G. Brunaccini , V. Antonucci , M. Ferraro , Energy Convers. Manage. 2020, 203, 112228;

[6] a) S. A. Tahami , M. Gholikhani , R. Nasouri , S. Dessouky , A. Papagiannakis , Appl. Energy 2019, 238, 786;

[7] G. J. Snyder , E. S. Toberer , Nat. Mater. 2008, 7, 105. 18219332 10.1038/nmat2090

[8] a) H. Luo , T. Yang , X. Jing , Y. Cui , W. Qin , Mater. Today Energy 2024, 101502;

[9] a) X. He , C. Li , S. Zhu , J. Cai , G. Yang , Y. Hao , Y. Shi , R. Wang , L. Wang , X. Li , Chem. Eng. J. 2024, 490, 151470;

[10] a) W. W. Che , C. Y. Tso , L. Sun , D. Y. Ip , H. Lee , C. Y. Chao , A. K. Lau , Energy Build. 2019, 201, 202;

[11] a) P. W. Tien , S. Wei , J. K. Calautit , J. Darkwa , C. Wood , Appli. Energy 2022, 308, 118336;

[12] X. Zhou , J. Carmeliet , D. Derome , Build. Environ. 2020, 175, 106773.

[13] a) M. Sun , Z. Li , Q. Mao , D. Shen , Cem. Concr. Res. 1998, 28, 549;

[14] a) X. Liu , R. Jani , E. Orisakwe , C. Johnston , P. Chudzinski , M. Qu , B. Norton , N. Holmes , J. Kohanoff , L. Stella , Renewable Sustainable Energy Rev. 2021, 137, 110361;

[15] a) E. Rausch , M. V. Castegnaro , F. Bernardi , M. C. M. Alves , J. Morais , B. Balke , Acta Mater. 2016, 115, 308;

[16] P. Hotěk , L. Fiala , R. Černý , J. Phys. Conf. Ser. 2023.

[17] a) G. Furtos , L. Molnar , L. Silaghi‐Dumitrescu , P. Pascuta , K. Korniejenko , J. Nat. Fibers 2022, 19, 6676;

[18] a) H.‐D. Yun , K. Rokugo , Cold Reg. Sci. Technol. 2012, 78, 82;

[19] a) R. Ming , W. Yu , X. Zhao , Y. Liu , B. Li , E. Essah , R. Yao , Energy Build. 2020, 208, 109611;

[20] a) T. W. B. Riyadi , M. Effendy , B. R. Utomo , A. T. Wijayanta , Appl. Therm. Eng. 2023, 235, 121336;

[21] a) F. Song , L. Wu , S. Liang , Nanotechnology 2012, 23, 085401; 22293218 10.1088/0957-4484/23/8/085401

[22] P. Code , BSI 2005, 668, 659.

[23] H. U. Ahmed , A. A. Mohammed , A. S. Mohammed , J. Build. Eng. 2022, 49, 104062.

[24] C. Ma , B. Zhao , S. Guo , G. Long , Y. Xie , J. Clean. Prod. 2019, 220, 188.

[25] B. Nematollahi , J. Sanjayan , F. U. A. Shaikh , Composites, Part B 2016, 89, 253.

[26] D. Zheng , W. Song , J. Fu , G. Xue , J. Li , S. Cao , Constr. Build. Mater. 2020, 258, 120351.

[27] B. Zhu , J. Pan , J. Li , P. Wang , M. Zhang , Cem. Concr. Compos. 2022, 133, 104677.

[28] a) K. H. Lee , S. I. Kim , J. C. Lim , J. Y. Cho , H. Yang , H. S. Kim , Adv. Funct. Mater. 2022, 32, 2203852;

[29] T. Ji , S. Zhang , Y. He , X. Zhang , X. Zhang , W. Li , J. Build. Eng. 2021, 43, 103190.

[30] J. P. Heremans , V. Jovovic , E. S. Toberer , A. Saramat , K. Kurosaki , A. Charoenphakdee , S. Yamanaka , G. J. Snyder , Science 2008, 321, 554. 18653890 10.1126/science.1159725

[31] S. Wen , D. Chung , Carbon 2006, 44, 2130.

[32] A. Chen , Z. Zhao , D. Xu , X. Liu , X. Chen , Hydrometallurgy 2013, 136, 46.

[33] F. Werner , J. Appl. Phys. 2017, 122.

[34] T. M. Higgins , D. McAteer , J. C. M. Coelho , B. M. Sanchez , Z. Gholamvand , G. Moriarty , N. McEvoy , N. C. Berner , G. S. Duesberg , V. Nicolosi , ACS Nano 2014, 8, 9567. 25199042 10.1021/nn5038543

[35] M. Hlobil , K. Sotiriadis , A. Hlobilova , Cem. Concr. Res. 2022, 154, 106714.

[36] Y. Zhang , S. Zhang , C. Liu , O. Çopuroğlu , Cem. Concr. Res. 2024, 176, 107409.

[37] a) L. Wang , Q. Yao , W. Shi , S. Qu , L. Chen , Mater. Chem. Front. 2017, 1, 741;

[38] Y. Zhang , O. Çopuroğlu , Cem. Concr. Res. 2024, 176, 107396.

[39] a) C. Li , Z. Jiang , R. J. Myers , Q. Chen , M. Wu , J. Li , P. J. Monteiro , Cem. Concr. Compos. 2020, 111, 103630;

[40] a) S. Hanjitsuwan , S. Hunpratub , P. Thongbai , S. Maensiri , V. Sata , P. Chindaprasirt , Cem. Concr. Compos. 2014, 45, 9;

[41] J. Zhou , Z. Yu , Y. Lv , C. Wang , P. Hu , Y. Liu , Composites, Part A 2022, 163, 107195.

[42] Z. Deng , S. Zhang , Z. Deng , J. Clean. Prod. 2023, 426, 139200.

[43] a) S. I. Kim , K. H. Lee , H. A. Mun , H. S. Kim , S. W. Hwang , J. W. Roh , D. J. Yang , W. H. Shin , X. S. Li , Y. H. Lee , Science 2015, 348, 109; 25838382 10.1126/science.aaa4166

[44] a) S. Acharya , B. K. Yu , J. Hwang , J. Kim , W. Kim , Adv. Funct. Mater. 2021, 31, 2105008;

[45] a) G. Cheng , T.‐H. Chang , Q. Qin , H. Huang , Y. Zhu , Nano Lett. 2014, 14, 754; 24382314 10.1021/nl404058r

[46] N. Jaziri , A. Boughamoura , J. Müller , B. Mezghani , F. Tounsi , M. Ismail , Energy Rep. 2020, 6, 264.

[47] H. Yokota , K. Rokugo , N. Sakata , presented at *High Performance Fiber Reinforced Cement Composites* , Springer, Tokyo, Japan 2008.

[48] a) J. Wei , Y. Zhou , Y. Wang , Z. Miao , Y. Guo , H. Zhang , X. Li , Z. Wang , Z. Shi , Energy 2023, 265, 126398;

