# Supplementary material for: Intelligent Thermoelectric Sensing with Sustainable Strain‐Hardening Geopolymeric Composites
Source: Small Sci. 2025 Jan 22;5(3):2400520. doi: 10.1002/smsc.202400520 (PMC12245009; doi:10.1002/smsc.202400520)
Supplement: Supplementary file 1 — Supplementary Material [file SMSC-5-2400520-s001.pdf]

Intelligent Thermoelectric Sensing with Sustainable Strain-Hardening Geopolymeric Composites

Contents

SI1. Energy dispersive spectroscopy (EDS) 3
SI2. Isothermal calorimetric curve 4
SI3. Fractal theory 5
SI4. X-CT test 6
SI5. Raman spectra and XRD analysis 8
SI6. FIB-TEM analysis 9
SI7. Electrical conductivity 10
SI8. Mixing and molding process 12
SI9. Thermal conductivity measurement 13
SI10. Seebeck coefficient measurement 15
SI11. Electrical conductivity measurement 16

SI1. Energy dispersive spectroscopy (EDS)

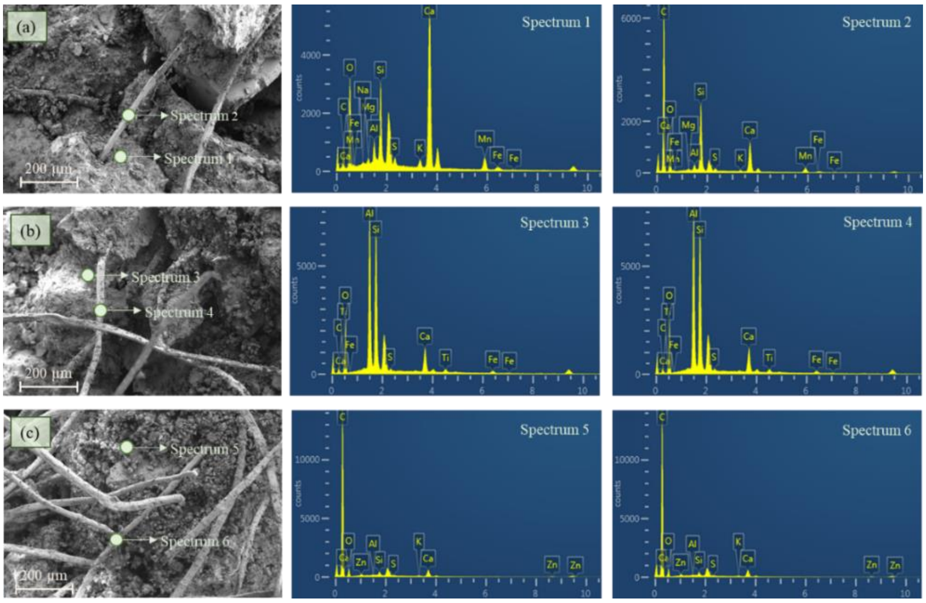

Figure S1. The SEM-EDS images of a mold-cast-SHGC matrix made using oxide admixture.

Table S1 | Elements related to (EDS)

Element	Spectrum 1 wt.%	Spectrum 2 wt.%	Spectrum 3 wt.%	Spectrum 4 wt.%	Spectrum 5 wt.%	Spectrum 6 wt.%
C	14.76	67.43	16.51	79.10	15.83	85.81
O	43.24	20.76	41.51	15.29	11.70	10.52
Na	0.32	0	0	0	0.23	0
Mg	0.65	0.12	0.32	0	0.57	0
Al	1.84	0.40	3.13	0.04	1.13	0.09
Si	7.04	4.84	10.52	0.83	4.79	0.53
S	0.99	0.27	1.26	0	0.43	0.15
K	0.98	0.16	0.71	0.10	0.39	0.15
Ca	24.60	4.23	21.81	2.78	39.97	2.03
Mn	4.65	1.60	0	0	0.37	0
Fe	0.94	0.20	4.24	1.86	21.13	0
Zn	0	0	0	0	3.45	0.72
Total:	100.00	100.00	100.00	100.00	100.00	100.00

The EDS data for the three different gel product morphologies of SHGCs are displayed in **Figure S1** and **Table S1.** The fundamental constituent elements were Si, O, Al, K, Ca, C, and Fe. The iron content in GFe-2-5 is nearly twice that of the other two samples. Additionally, Mn was detected in GMn-2-5, while Zn was identified in GZn-2-5. It can be clearly observed that the carbon content of the fibers is higher than others. Note that the calculation error of EDS for Ca, Si, and O is within 5%, while the error for other elements is within 10%.

SI2. Isothermal calorimetric curve

In order to investigate the role of additives in geopolymerization reactions, this study conducted micro-isothermal calorimetry experiments with different additives. The heat release rate curves exhibit two distinct peaks: an initial peak and a second peak (**Figure 2a**). The initial peak, observed during the first few minutes, is significantly greater than the second peak. For the one-part geopolymer composite samples (GCs), the time of the second peak varied, occurring approximately between 20 and 36 hours.

Notably, samples with ZnO did not exhibit a second peak, which might be attributed to the interaction.[1] The initial products of the reaction include zincate ions, which may subsequently yield sodium zinc silicate under the conditions of the silicate reaction system. Upon the addition of water, the solid activator rapidly dissolves, resulting in a significant release of thermal energy and the formation of a highly concentrated solution containing OH^- ions and $[SiO_4]^{4-}$ ions.[2] Following that, the reaction process transitions into the second stage, called the induction period. During this interval, the concentration of Ca^{2+} increases and more oligomers are formed, despite the sluggish reaction rates.[3] There is a great increase in cumulative heat release throughout the early dissolution, induction, and acceleration periods, which occur during the first several hours (**Figure 2b**).

Meanwhile, the 48-hour cumulative heat release of GMn-5-P is the highest, whereas that of GZn-5-P is the lowest. The sample GMn-5-P exhibited an earlier peak time compared to G-0-P, which could be attributed to the catalytic effect of MnO_2.[4] The behaviour of sample GFe-5-P can be explained by the partial reaction between alkali and iron oxide.[5]

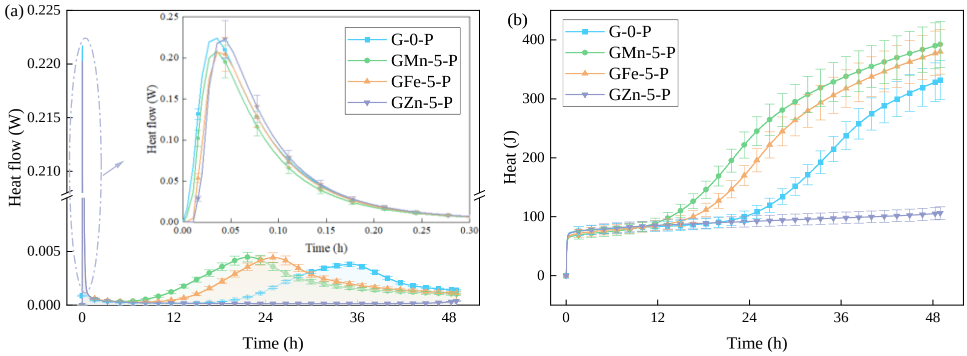

Figure S2. Isothermal calorimetric reaction a) Geopolymerization reaction process of heat flow curves, which has an uncertainty of about ±10%. b) Geopolymerization reaction process and

mechanical properties of heat curves, which has an uncertainty of about ±10%.

SI3. Fractal theory

Fractal theory facilitates the quantitative analysis of complex phenomena in the physical realm. This approach has gained extensive application in rock mechanics and concrete material studies in recent years.[6] The box-counting method is commonly employed to assess the fractal dimension of crack formations.[7] N_r represents the corresponding total number of boxes, while r denotes square grids of side length. The fractal dimension (D) could be calculated by **equation 1**:

$$D = -\frac{lnN_r}{lnr} \tag{1}$$

MATLAB software was employed for digital image analysis with grayscale conversion. Therefore, the $ln(r)\ -\ ln\ (N_r)$ curves of the dog bone specimens exhibit linear relationships, suggesting that the distribution of surface cracks follows a statistical self-similarity structure.

SI4. X-CT test

An industrial CT scanner with high resolution (YXLON) was used to analyse the microstructure on 50-mm cast GC or SHGC specimens. Avizo software scanned and reconstructed the test samples, producing images with dimensions of 1024×1024 pixels, spaced 0.1 mm apart vertically per pixel. This research used greyscale values (GSV) of the ROI from XCT scanning to look at the structure of the pores in GCs and SHGCs. We used the open-source image processing program ImageJ for the pre-treatment. The hardened GC and SHGC samples display an obvious contrast in gray level between the porous and solid phases due to the distinct physical densities. A fixed threshold of 30 (grey level) was selected to distinguish between pore and solid phases, which relied on the grey-level histogram.[8]

The matrix randomly distributed irregularly ellipsoid-shaped natural pores, some of which had small pores attached to their larger surfaces. In addition to visual observation, we can obtain the volume and size of each pore (**Figure S3g**). The frequency histograms of pore volume are presented to quantify the pore size distribution. The matrix of GC is dense, and hydration is adequate. However, samples with PVA fibers are characterised by numerous pores, which can facilitate the propagation of cracks, leading to lower fracture toughness and initially lower crack resistance strength. This promotes saturation and multiple cracking behaviours in SHGCs.[9] The study focused on pores bigger than 10 μm^3 in the matrix for convenient statistical analysis. **Figure S3** separately catalogues the pore structures ranging from 10-1000 μm^3 and those exceeding 1000 μm^3. The GMn-5-P sample exhibited a higher number of larger pores compared to the G-0 and GMn-5 samples without PVA fibers. For SHGC samples, the porosity initially decreased and then increased with increasing MnO_2 content. Regarding the SHGC samples added with different oxides, the number of pores larger than 1000 μm^3 followed the order: GMn-5-P, GZn-5-P, GFe-5-P.

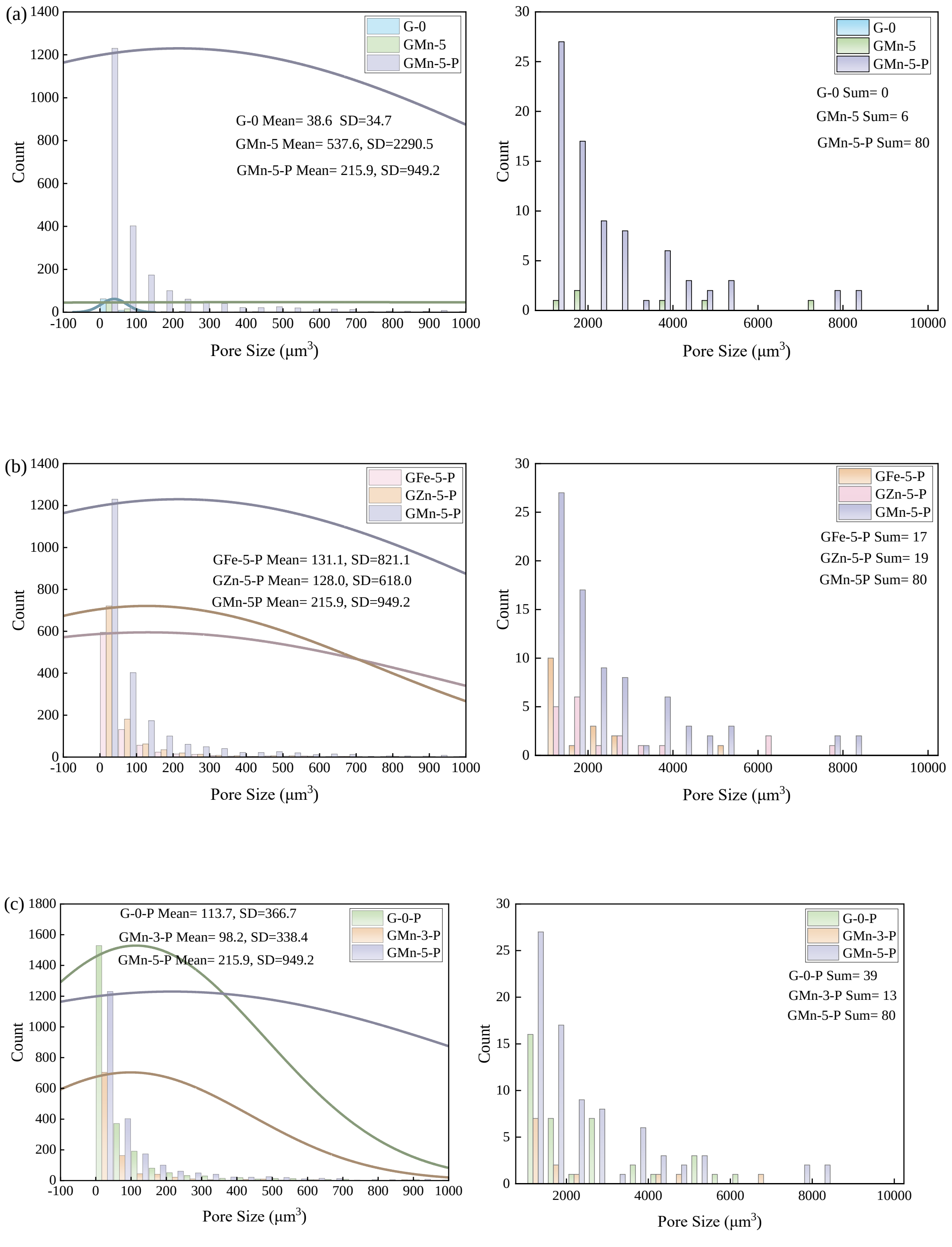

Figure S3. The X-CT images a-c) Frequency histogram and Cumulative frequency distribution diagram of small pores.

SI5. Raman spectra and XRD analysis

The Raman active vibrations of the C=C double bonds are located at 1500 cm^{-1}, where the symmetric stretching vibrations of the π electron clouds result in this characteristic peak.[10] However, the GFe-5-P sample does not display these bands. In the samples GMn-5 and GMn-5-P, the broad bands centered at about 640 cm^{-1} may be related to the Mn-O bond.[11] Additionally, the significantly higher intensity of the signal around 470 cm^{-1} observed in GFe-5-P is ascribed to the Fe-O bond.

Figures S4d and S4e display the reaction products of geopolymeric composites, as characterized by X-ray diffraction. The major peak is observed at around 25°, which differs from the peaks observed in cement mortar. The change in the major peak can be classified as geopolymerization. Adding quartz sand caused some diffraction peaks to resemble quartz. Calcite peaks were also detected, consistent with recent findings that $CaCO_3$ crystals are a primary reaction product in the medium to long term.[12] In sample GFe-5-P, a greater number of sharp diffraction peaks were observed.

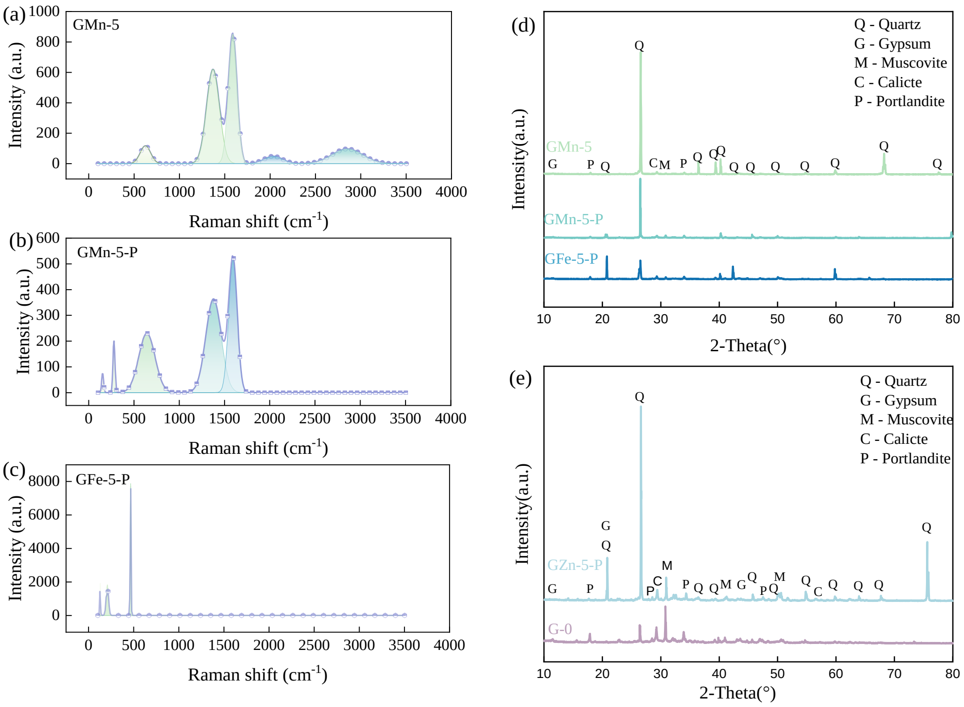

Figure S4. The Raman spectrum and X-ray diffraction curve.

SI6. FIB-TEM analysis

Additionally, the polished surfaces were characterised using TEM. The samples were used in a dual-beam FIB-SEM system (Zeiss Crossbeam 540) to precisely navigate to the manganese dioxide interfaces. This region exhibited clear manganese dioxide cementation with the matrix as indicated by the purple rectangle in **Figure S5a**. Subsequently, the surface was coated with platinum, and the matrix on both sides of the platinum coating was carefully milled using a focused gallium ion. The target region beneath the platinum cover was then extracted from the matrix and transferred to a Cu support (**Figure S5c**). The extracted slice was further thinned using a focused gallium ion beam to obtain a sample with a thickness of approximately 100 nm.

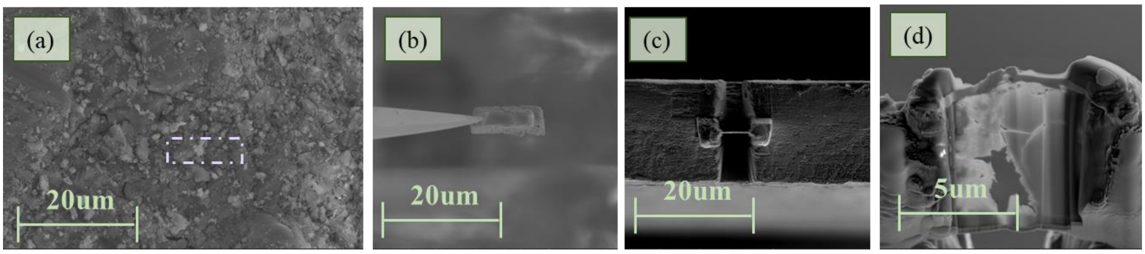

Figure S5. a) target area (purple box). b) extracting slice. c) placing on Cu support. d) milling for TEM analysis.

SI7. Electrical conductivity

Current can be given in two ways: through AC (alternating current) and DC (direct current).[13] Utilizing AC for resistance measurement aligns the dipoles of ions to establish a conductive route for electric current.[14] Geopolymer materials have a capacitive characteristic that allows them to store electrical charge. AC measurement considers a non-resistive element known as reactance, leading to the measurement of impedance rather than resistance.

GCs and SHGCs typically exhibit complex porous structures. Consequently, their resistivity values are contingent upon their pore sizes. In porous materials, there may exist two modes of conductivity, namely electronic and electrolytic conduction. Electronic conduction occurs due to the movement of free electrons along paths formed by conductive fibers. Electrolytic conduction, observed to be more pronounced in moist samples, results from the motion of ions within the porous structure.

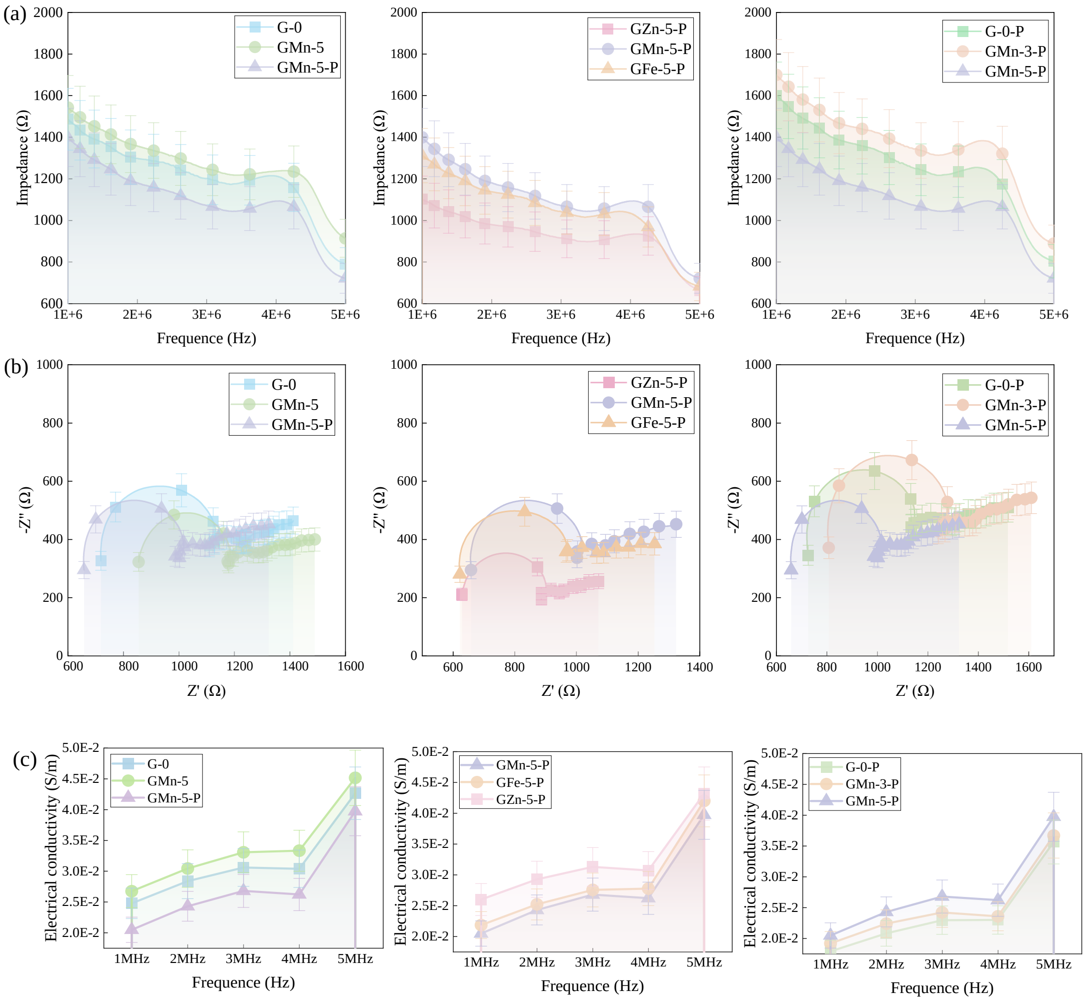

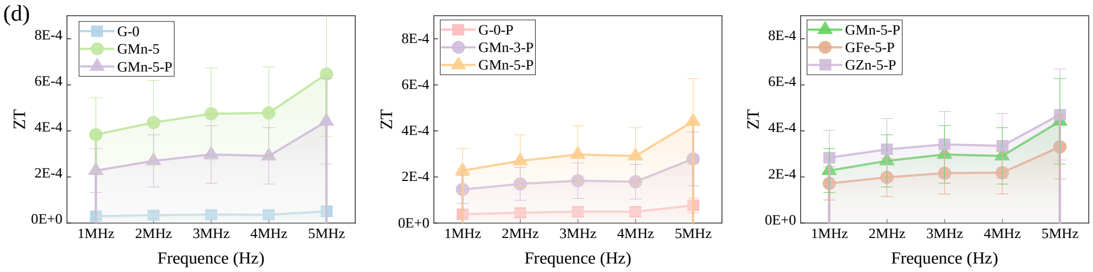

Figure S6. *ZT* value under AC impedance a) Frequency-dependent impedance modulus, which has an uncertainty of about ±10%. b) Nyquist plot, which has an uncertainty of about ±10%. c) Impedance at a specific frequency, which has an uncertainty of about ±10%. d) *ZT* value at a specific frequency, which has an uncertainty of about ±42%.

SI8. Mixing and molding process

The mixing and molding process is illustrated in **Figure S7.** In the composition of OP-SHGC, OPG powder, PVA fibers, fine aggregate, and water serve as the foundational materials. OPGC comprises a homogeneous mixture of fly ash and ground granulated blast furnace slag (GGBS), each holding an equal mass percentage. The chemical activation of this precursor is achieved

through the incorporation of sodium metasilicate powder and sodium silicate powder. These activators, mixed in equal proportions, contribute 10 wt.% of the mass to the precursor by prior studies.[15] Finally, type-W PVA fibers, sourced from Baohua Lin Industrial Development Co., Ltd (China), are incorporated into the mixture.

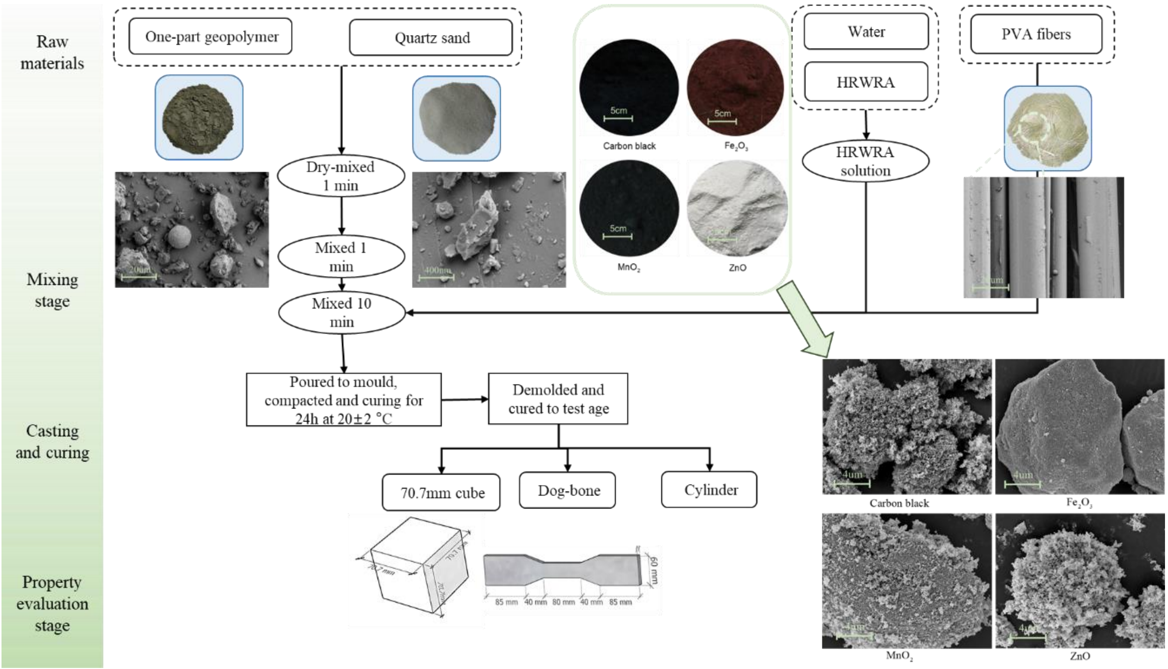

Figure S7. Schematic image of OPGC stirring and molding.

SI9. Thermal conductivity measurement

This study analyzed the thermal behavior using a self-designed device (**Figure S8**). Temperature monitoring was conducted using eight thermocouples (S1-S8) positioned strategically on the specimen. Data were recorded at two-second intervals, and a steady state was presumed once successive thermal conductivity readings deviated by less than 3%.

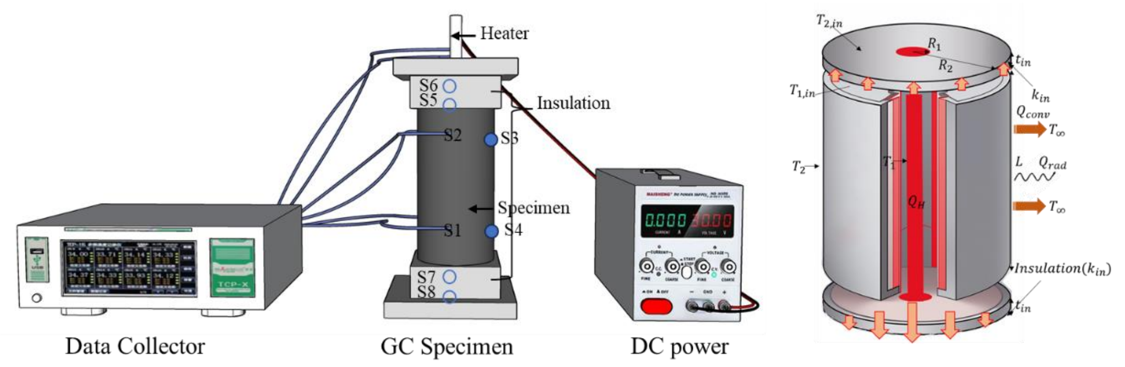

Figure S8. Thermal conductivity measurement: This setup included a cylindrical specimen with a central 12 mm diameter hole, into which a 10 mm heating rod was inserted. The gap around the rod was filled with silicone grease (thermal conductivity: 6 $\mathrm{Wm^{-1}K^{-1}}$) for efficient heat transfer. The specimen was sandwiched between two 20-mm thermal insulation layers (thermal conductivity: 0.033 $\mathrm{Wm^{-1}K^{-1}}$), secured by clamps. Heating was provided by a DC power source set at 9.5 W.

The temperature distribution mentioned above satisfies the Laplace equation and may be transformed into cylindrical coordinates, as seen below in **equation (2)**:

$$\frac{1}{r}\frac{\partial}{\partial r}\left(r\frac{\partial T}{\partial r}\right)+\frac{1}{r^2}\frac{\partial^2 T}{\partial \theta^2}+\frac{\partial^2 T}{\partial z^2}=0 \tag{2}$$

When the heat conduction mechanism exhibits horizontal symmetry and the z-direction component is zero as a result of axial symmetry. **Equation (3)** may be represented as

$$\frac{d}{dr}\left(r\frac{dT}{dr}\right)=0 \tag{3}$$

Equation (3) can be formulated based on Fourier's law and boundary conditions, as shown in **Equations (4-7).**

$$\dot{Q}_{cond}-2\pi kL\frac{T_1-T_2}{\ln\left(\frac{r_2}{r1}\right)}=0 \tag{4}$$

$$\dot{Q}_{cond}=\dot{Q}_{heat}-\dot{Q}_{loss} \tag{5}$$

$$\dot{Q}_{loss}=k_{in}A\frac{T_{1,in}-T_{2,in}}{t_{in}} \tag{6}$$

$$\dot{Q}_{heat}=V\times I \tag{7}$$

By taking into account the energy loss and utilizing the simplified **Equations (5-7)**,[16] **Equation 4** can be solved.

SI10. Seebeck coefficient measurement

To ensure consistent measurements, each specimen was carefully prepared and uniformly polished. A thin layer of silver pastes and copper adhesive facilitated the recording of TE voltages across the samples (**Figure S9**). A small resistance heater (JF-976S, Jinfeng Electronics, Dongguan) was utilized to create a temperature gradient, and the temperatures were precisely monitored using a multi-channel temperature measuring instrument (XCP-XL Huipu Co., Ltd.). The voltage differential between the copper connections was continuously monitored with a KEITHLEY DIGIT Multimeter (DMM 7510), interfaced with KickStart Software, ensuring accurate data acquisition and analysis, and the Seebeck Coefficient of the wires was calibrated.

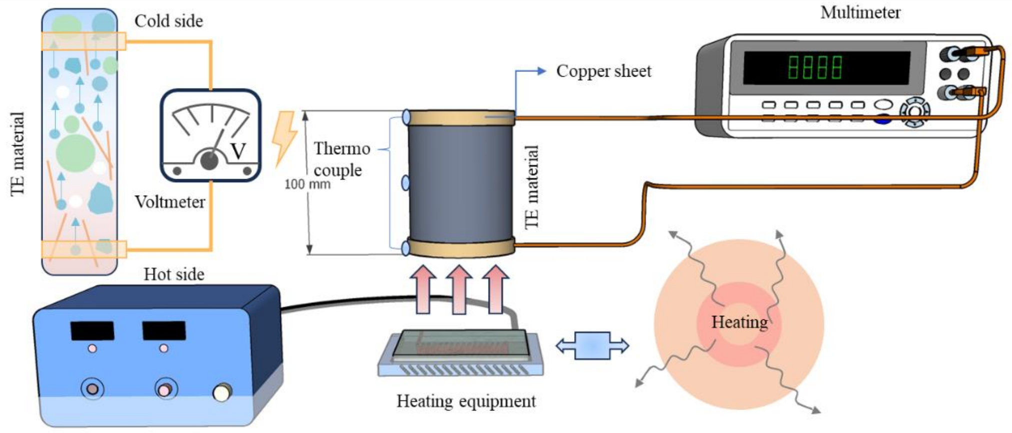

Figure S9. Seebeck coefficient measurement.

SI11. Electrical conductivity measurement

AC impedance spectroscopy was performed using a HIOKI 3570 impedance analyser. Data were recorded across a frequency range of 10 Hz to 5 MHz, with a voltage amplitude set at 1 V. From these impedance data, the AC conductivity of the samples was calculated and reported. Additionally, the DC resistance was analyzed using the LCR program. The samples used copper disc electrodes with a diameter of 50 mm, which facilitated data acquisition (**Figure S10**).

For DC conductivity, the article utilized specific impedance to quantify the conductivity of the specimen, which was mathematically represented by **equation (8)**.

$$\sigma = \frac{L}{|Z|A} \tag{8}$$

where σ is the specific impedance (Ω·m), $|Z|$ is the total impedance (Ω), A is the specimen cross-sectional area (m^2), and L is the specimen effective length (m).

The electrical conductivity can be calculated by multiplying the carrier density n and the elementary charge e by the mobility μ, as seen in **equation (9)**.

$$\sigma = ne\mu = ne^2\tau/m^* \tag{9}$$

where τ represents the energy-independent relaxation time and m^* is the effective mass of the carrier. It is important to note that discrepancies between Hall measurements and our electrical conductivity measurements can be attributed to the small size of the samples tested. Considering the inherent microscale heterogeneity of these materials, inconsistencies in data are not uncommon. Such differences are also influenced by variations in measurement setups and conditions. However, we focus primarily on the observed trend rather than absolute values.

Additionally, measuring the TE properties of geopolymers presents significant challenges due to their heterogeneous nature and often low conductivity. These challenges emphasize the necessity for more rigorous and standardized methods in the future to ensure that data remains consistent and reliable.

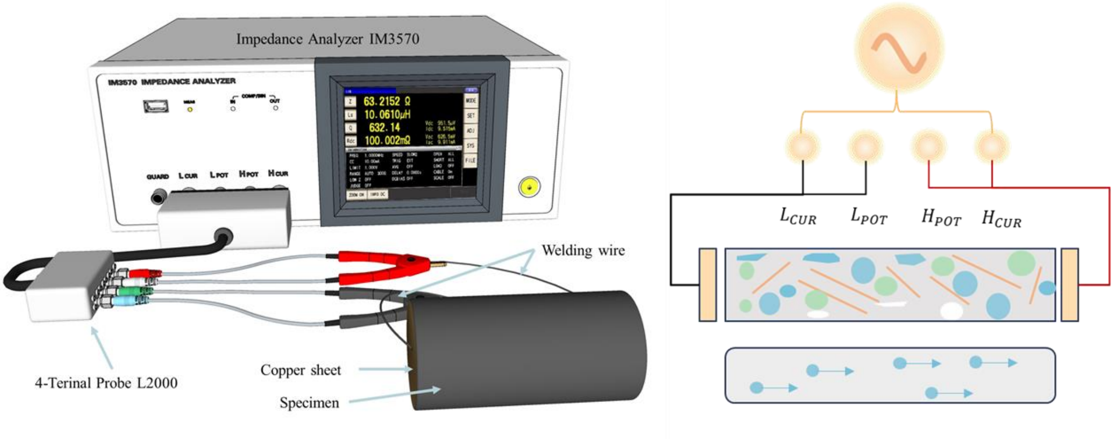

Figure S10. Electrical conductivity measurement.

References

[1] A. Chen, Z. Zhao, D. Xu, X. Liu, X. Chen, *Hydrometallurgy* **2013**, 136, 46.

[2] M. B. Haha, B. Lothenbach, G. Le Saout, F. Winnefeld, *Cement Concrete Res* **2012**, 42, 74.

[3] a)Q. Wan, F. Rao, S. Song, R. E. García, R. M. Estrella, C. L. Patiño, Y. Zhang, *Cement and Concrete Composites* **2017**, 79, 45; b)W. Hu, Q. Nie, B. Huang, X. Shu, *J Clean Prod* **2019**, 215, 1481.

[4] P. Wei, G. Yin, M. Shi, J. Zhang, Z. Yang, J. Feng, presented at *IOP Conference Series: Earth and Environmental Science*, **2021**.

[5] C. Siakati, A. P. Douvalis, V. Hallet, A. Peys, Y. Pontikes, *Cement Concrete Res* **2021**, 146, 106466.

[6] a)S. Erdem, M. A. Blankson, *Constr Build Mater* **2013**, 40, 70; b)M. Armandei, E. de Souza Sanchez Filho, *Constr Build Mater* **2017**, 140, 82; c)L. Restuccia, A. Reggio, G. Ferro, R. Kamranirad, *Theoretical and Applied Fracture Mechanics* **2017**, 90, 133; d)D. Zheng, W. Song, J. Fu, G. Xue, J. Li, S. Cao, *Constr Build Mater* **2020**, 258, 120351.

[7] A. Rezaie, A. J. Mauron, K. Beyer, *Automation in Construction* **2020**, 117, 103258.

[8] J. Ye, C. Cui, J. Yu, K. Yu, J. Xiao, *Composites Part B: Engineering* **2021**, 211, 108639.

[9] B. Zhu, J. Pan, J. Li, P. Wang, M. Zhang, *Cement and Concrete Composites* **2022**, 133, 104677.

[10] a)A. V. Soldatova, G. Balakrishnan, O. F. Oyerinde, C. A. Romano, B. M. Tebo, T. G. Spiro, *Environmental science & technology* **2019**, 53, 4185; b)V. E. de Oliveira, H. V. Castro, H. G. Edwards, L. F. C. de Oliveira, *Journal of Raman Spectroscopy* **2010**, 41, 642.

[11] a)M. C. Caggiani, A. Coccato, G. Barone, C. Finocchiaro, M. Fugazzotto, G. Lanzafame, R. Occhipinti, A. Stroscio, P. Mazzoleni, *Journal of Raman Spectroscopy* **2022**, 53, 617; b)E. Vermeersch, F. Košek, J. De Grave, J. Jehlička, P. Vandenabeele, A. Rousaki, *Journal of Raman Spectroscopy* **2023**, 54, 1201.

[12] A. F. Abdalqader, F. Jin, A. Al-Tabbaa, *J Clean Prod* **2016**, 113, 66.

[13] I. Hansson, C. Hansson, *Cement Concrete Res* **1983**, 13, 675.

[14] a)H. Layssi, P. Ghods, A. R. Alizadeh, M. Salehi, *Concrete international* **2015**, 37, 41; b)S. R. Armoosh, M. Oltulu, presented at *IOP Conference Series: Materials Science and Engineering*, **2019**.

[15] Y. Yuan, B. Ding, H. Shi, Y. Wang, Y. Lin, M. Elchalakani, J. Cai, *J Build Eng* **2024**, 91, 109538.

[16] J. Carlson, R. Bhardwaj, P. Phelan, K. Kaloush, J. Golden, *J Mater Civil Eng* **2010**, 22, 186.
